# Phentolamine Significantly Enhances Macrolide Antibiotic Antibacterial Activity against MDR Gram-Negative Bacteria

**DOI:** 10.3390/antibiotics12040760

**Published:** 2023-04-14

**Authors:** Ze-Hua Cui, Hui-Ling He, Zi-Jian Zheng, Zhao-Qi Yuan, Ying Chen, Xin-Yi Huang, Hao Ren, Yu-Feng Zhou, Dong-Hao Zhao, Liang-Xing Fang, Yang Yu, Ya-Hong Liu, Xiao-Ping Liao, Jian Sun

**Affiliations:** 1 Guangdong Laboratory for Lingnan Modern Agriculture, National Risk Assessment Laboratory for Antimicrobial Resistance of Animal Original Bacteria, College of Veterinary Medicine, South China Agricultural University, Guangzhou 510642, China; 2 Guangdong Provincial Key Laboratory of Veterinary Pharmaceutics, Development and Safety Evaluation, South China Agricultural University, Guangzhou 510642, China; 3 Jiangsu Co-Innovation Center for the Prevention and Control of Important Animal Infectious Diseases and Zoonoses, Yangzhou University, Yangzhou 225009, China

**Keywords:** Gram-negative pathogens, phentolamine, macrolide antibiotics, synergistic antibacterial activity

## Abstract

Objectives: Multidrug-resistant (MDR) Gram-negative bacterial infections have limited treatment options due to the impermeability of the outer membrane. New therapeutic strategies or agents are urgently needed, and combination therapies using existing antibiotics are a potentially effective means to treat these infections. In this study, we examined whether phentolamine can enhance the antibacterial activity of macrolide antibiotics against Gram-negative bacteria and investigated its mechanism of action. Methods: Synergistic effects between phentolamine and macrolide antibiotics were evaluated by checkerboard and time–kill assays and in vivo using a *Galleria mellonella* infection model. We utilized a combination of biochemical tests (outer membrane permeability, ATP synthesis, ΔpH gradient measurements, and EtBr accumulation assays) with scanning electron microscopy to clarify the mechanism of phentolamine enhancement of macrolide antibacterial activity against *Escherichia coli*. Results: In vitro tests of phentolamine combined with the macrolide antibiotics erythromycin, clarithromycin, and azithromycin indicated a synergistic action against *E. coli* test strains. The fractional concentration inhibitory indices (FICI) of 0.375 and 0.5 indicated a synergic effect that was consistent with kinetic time–kill assays. This synergy was also seen for *Salmonella typhimurium*, *Klebsiella pneumoniae*, and *Actinobacter baumannii* but not *Pseudomonas aeruginosa*. Similarly, a phentolamine/erythromycin combination displayed significant synergistic effects in vivo in the *G. mellonella* model. Phentolamine added singly to bacterial cells also resulted in direct outer membrane damage and was able to dissipate and uncouple membrane proton motive force from ATP synthesis that, resulted in enhanced cytoplasmic antibiotic accumulation via reduced efflux pump activity. Conclusions: Phentolamine potentiates macrolide antibiotic activity via reducing efflux pump activity and direct damage to the outer membrane leaflet of Gram-negative bacteria both in vitro and in vivo.

## 1. Introduction

Antimicrobial resistance in animal and human pathogens represents a major global health crisis since the prevalence of multidrug-resistant (MDR) clinical bacterial isolates are currently on the rise [1,2]. The World Health Organization’s priority list of resistant bacteria includes three Gram-negative species at the critical ranking, the highest level of concern [1]. Annual deaths due to increased antimicrobial resistance are predicted to reach 10 million by 2050. Importantly, MDR Gram-negative infections have limited treatment options due, and new therapeutic strategies and agents must be developed to overcome these treatment barriers. Unfortunately, the global rise in MDR infections has been accompanied by a failure in antibacterial drug discovery, and few antibiotics in clinical use over the past 40 years have been novel compounds; most have originated from known chemical structures [2].

In spite of advances in genomics and high-throughput screening technologies, structurally and mechanistically distinct antibiotic classes outside the common targets have not been developed, and other approaches are required [3]. One practical strategy is the use of drug combinations, such as the use of β-lactamase inhibitors in combination with carbapenems for the treatment of carbapenem-resistant Gram-negative bacteria [4].

Aerobic Gram-positive and Gram-negative cocci, *Legionella*, *Mycoplasma,* and *Chlamydia,* as well as some anaerobic infections, have traditionally been treated with macrolides and their lactone derivatives. However, macrolide administration is also associated with extensive pharmacological (side) effects. For example, erythromycin was the first macrolide antibiotic and was discovered in soil samples in 1949 [5], but these macrolides (especially erythromycin and clarithromycin) have poor antibacterial effects on Gram-negative pathogens due to outer membrane impermeability coupled with promiscuous efflux pumps. High-throughput screening of natural products and synthetic compounds has uncovered some additional antibacterial effects for macrolides against Gram-positive pathogens. Unfortunately, these drugs are similar to erythromycin in that they cannot either enter or accumulate within Gram-negative pathogens [3,6]. Therefore, combination therapy with existing antibiotics is a feasible option to overcome these particular problems [7]. In the ideal scenario, compounds that cannot penetrate the Gram-negative cell envelope can be used in combination with membrane penetrators or efflux pump inhibitors. This is a promising strategy for combating Gram-negative bacterial infections.

Phentolamine is a reversible, nonselective α-adrenergic antagonist, and its primary application is the control of hypertensive emergencies and pheochromocytoma in particular [8]. This compound has been successfully used to manage cocaine-induced cardiovascular complications and, importantly, to counteract severe peripheral vasoconstriction and pain due to infusions of vasopressors such as norepinephrine. Phentolamine has also been successfully used as a diagnostic and therapeutic agent to treat complex regional pain syndrome [9,10,11]. Our laboratory has found that phentolamine can greatly improve the antibacterial activity of erythromycin against *Escherichia coli* (unpublished data). In the current study, we examined whether phentolamine can enhance the antibacterial activity of macrolide antibiotics against Gram-negative bacteria and determined its mechanism of action in this process.

## 2. Materials and Methods

### 2.1. Bacterial Strains and Culture Conditions

A clinical *E. coli* isolate 18FS from duck feces was chosen for this study and was identified to the species level using MALDI-TOF MS (Shimadzu, Kyoto, Japan). All other bacterial strains were purchased from the American Type Culture Collection (Manassas, VA, USA).

A *tolC* knockout in *E. coli* BW25113 was constructed using the pCasKP-apr plasmid electroporated into BW25113 to construct a BW25113/pCasKP strain. The *tolC::kan* 1527 bp fragment was then amplified from the PKD4 plasmid by PCR amplification with the primers *tolC*F and *tolC*R. The *tolC::kan* fragment was purified and electroporated into BW25113/pCasKP to generate a *tolC* gene knockout strain, and transformants were selected on Luria Bertani (LB) agar plates containing 30 mg/L kanamycin. The *tolC* gene disruption was confirmed by PCR using upstream and downstream primers of the *tolC* gene (Table S1 from Supplementary).

### 2.2. Antimicrobial Agents

Erythromycin (ERY), clarithromycin (CLA), and azithromycin (AZM) were purchased from Yuan Ye Biological Technology (Shanghai, China). Phentolamine was purchased from Maklin (Shanghai, China). ERY and AZM were dissolved in ethanol, CLA was dissolved in methanol, and all concentrations were 5120 mg/L. Phentolamine was dissolved in water as a 10 mM stock solution and diluted to the required working concentrations depending on the assay type.

### 2.3. MIC Determinations and Fractional Inhibitory Concentration (FIC) Index Assay

Antimicrobial susceptibility assays using the microdilution broth method were performed and interpreted according to CLSI guidelines (CLSI, 2018). The minimal inhibitory concentration (MIC) of the macrolide antibiotics for each test strain was determined in the presence of 2-fold increasing phentolamine concentrations (1/4–1/2 MIC) using a modified broth microdilution method as previously reported [12]. *Staphylococcus aureus* ATCC 29213 served as the quality control strain.

The Fractional Inhibitory Concentration Index (FICI) was determined using the chequerboard method, as previously reported for nitrofurantoin/amikacin combinations [13]. Briefly, serial dilutions of phentolamine and erythromycin in 96 well plates were inoculated with 5 × 10^5^ CFU/mL test bacteria and incubated for 18 h at 37 °C. Bacterial growth was determined using light scattering at 600 nm. The FICI was then calculated as previously described [14], and FICI ≤ 0.5 represents synergy and FICI ≥ 4 represents antagonism.

### 2.4. In Vitro Time–Kill Curves

Time–kill experiments were conducted to further characterize the synergistic activity of the phentolamine and macrolide combinations as previously described [15]. In brief, ~10^6^ colony-forming units (CFU)/mL of logarithmic phase cells were incubated with erythromycin in the presence and absence of phentolamine. Samples were taken at 0, 3, 6, 9, and 24 h following incubation at 37 °C, and CFU counts on Mueller–Hinton agar (MHA) plates were used to assess killing efficiency. Synergy was defined as achieving a ≥ 2 log_10_ CFU/mL reduction at 24 h in a combination versus the most active individual drug concentration [16]. The experiments were replicated independently three times.

### 2.5. Galleria mellonella Infection Model

The *G. mellonella* model system was used to assess in vivo antibiotic functions, as we previously reported [15]. In brief, *G. mellonella* larvae were obtained from Kaide Ruixin (Tianjin, China), and the optimal infective dose was determined using 250 mg cream-colored larvae that we randomly distributed into six experimental groups (n = 10/group). The larvae were infected with 10 μL injections containing 10^6^ CFU logarithmic-phase bacteria in the distal left proleg. The larvae were then transferred to plastic Petri dishes and incubated at 37 °C for 72 h and scored daily for survival. PBS injections served as negative controls. The in vivo efficacy of phentolamine and erythromycin, each used singly and in combination, resulting in an optimal infective dose of ~10^4^ CFU/larvae. The animals were randomized at 2 h post-infection (hpi) to receive no therapy or phentolamine and erythromycin either singly or in combination (n = 10/group). Antibiotics (10 μL) were administered once into the distal right proleg using phentolamine at 20 µM/larvae and 30 mg/kg erythromycin [17]. The animals were observed daily, and the percent survival was calculated for each group.

### 2.6. SEM Analysis

Mid-logarithmic phase *E. coli* cells (1 × 10^8^ CFU/mL) were treated with phentolamine (1–4 mM) in 20 mL culture medium at 37 ℃ for 1.5 or 3 h, and suspensions were centrifuged and washed 3 × with PBS. The resulting pellets were fixed with 1.5 mL 2.5% glutaraldehyde at 4 °C overnight. Samples were washed 3 × with PBS and then dehydrated using a 30, 50, 70, 90, and 95% ethanol series. Sample aliquots were applied to copper tape, air-dried, and sputter-coated. SEM analysis was performed at Shinake Biotechnology (Guangzhou, China).

### 2.7. Outer Membrane Permeability

Uptake of phenylnaphthylamine (NPN) (Meilunbio, China) was used to assess outer membrane permeability as previously described [12]. Briefly, bacteria (1 × 10^8^ CFU/mL) were washed 3 × with PBS and suspended in 0.5, 1, 2, and 4 mM phentolamine solutions for 30 min, then centrifuged and resuspended in PBS containing NPN for 30 min. The samples were again centrifuged, pellets were suspended in PBS, and absorbance was monitored using a Multimode Plate Reader (Perkin Elmer, China) in a 96-well plate format.

### 2.8. Intracellular pH Assay

Stationary phase bacterial cells were washed 3 × with PBS and suspended to an OD_600_ nm in PBS, followed by the addition of 3 µM (final) BCECF-AM (2’,7’-Bis-(2-carboxyethyl)-5-(and-6)-carboxy-fluorescein acetoxymethyl ester). The samples were then added to 96-well plates and incubated at 37 °C for 10 min. Phentolamine was then added to final concentrations of 0.5–8 mM, and sample fluorescence was monitored over the course of 1 h at excitation and emission wavelengths of 488 and 535 nm, respectively, in a Multimode Plate Reader (Perkin Elmer).

### 2.9. Intracellular ATP

Intracellular ATP levels in *E. coli* strain BW25113 were determined using an enhanced ATP Assay Kit using the protocol of the manufacturer (Beyotime, China). In brief, an *E. coli* BW25113 overnight culture was washed and suspended to OD_600_ nm = 0.5. Phentolamine (0.125–1 mM) was then added, and the cultures were incubated at 37 °C for 3 h. The cells were then pelleted by centrifugation, and the supernatants were discarded. The cell pellets were lysed with lysozyme and centrifuged. The supernatants were added to the detecting solution in a 96-well plate that was then incubated at room temperature for 5 min. Supernatant luminescence was measured using the Multimode Plate Reader (Perkin Elmer), and intracellular ATP levels were calculated from the luminescence data.

### 2.10. EtBr Accumulation Assay

*E. coli* cells were cultured to OD_600_ nm of 0.6 and pelleted by centrifugation (5000 rpm, 5 min), and washed 3 × with PBS. Phentolamine was added to separate cultures at 8, 4, 2, 1, and 0.5 mM. The suspensions (190 μL) were added to 96-well plates, and EtBr (10 μL) was added to 25 µM. The fluorescence of the cultures was then measured at excitation and emission wavelengths of 520 and 600 nm, respectively.

### 2.11. Statistical Analysis

Bacterial CFU were analyzed following log transformation in Prism 7.0 (GraphPad, San Diego, CA, USA). Two-tailed Mann–Whitney U-tests were used to calculate *p* values, and *p* ≤ 0.05 was considered significant. All data were presented as mean ± SD.

## 3. Results

### 3.1. In Vitro Interaction Assessments and Time–Kill Curves

**Figure 1:**
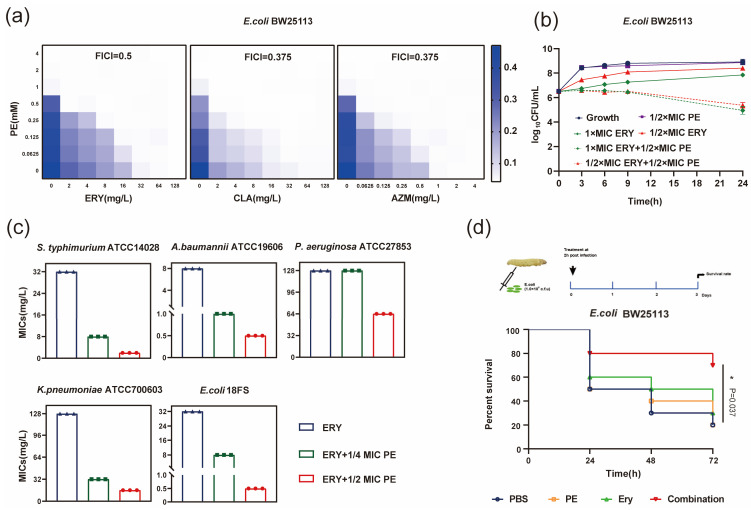
Potentiation of phentolamine (PE) in combination with the indicated macrolide antibiotics against test strains. (**a**) Checkerboard assay results for the indicated macrolides are shown as 8 × 8 matrix heat map graphs, and blue color intensity gradients represent bacterial cell densities (OD_600_). (**b**) Time–kill curves for *E. coli* BW25113 over 24 h following exposure to PE (1/2×MIC) in the presence of increasing ERY. (**c**) MICs of ERY for different species of Gram-negative bacteria exposure to PE (1/4-1/2×MIC). (**d**) Therapeutic effects of phentolamine combined with erythromycin in the *G. mellonella* model. Larval survival for phentolamine and erythromycin alone and in combination. * *p* < 0.05.

We selected an *E. coli* model strain BW25113 to compare with clinical isolate 18FS to evaluate interactions between phentolamine and the macrolides ERY, CLA, and AZM using a checkerboard assay format. Strain 18FS displayed resistance to a variety of antibiotics tested, including gentamicin, florfenicol, and tetracycline, and was, thus, classified as MDR (Table S2 from Supplementary). Incubation of strains BW25113 and 18FS with phentolamine in the presence of ERY, CLA, and AZM resulted in FICI values from 0.375 to 0.5, denoting synergism (Figure 1a; Figure S1a from Supplementary). Kinetic time–kill assays for these strains were then used to evaluate the pharmacodynamics of this effect. In vitro, bactericidal activities for all 3 macrolides against strain BW25113 were increased in the presence of phentolamine. The antibiotic-phentolamine combinations resulted in excess of 2 log_10_ CFU/mL reductions in bacterial densities, again indicating synergy (Figure 1b; Figure S1b from Supplementary). Interestingly, the MDR strain 18FS displayed reduced cell densities of exceed 4 log_10_ CFU/mL in the antibiotic–phentolamine combinations indicating a strong synergistic effect. Remarkably, CLA- and AZM-phentolamine combinations killed all the test bacteria and were most likely due in part to strain specificity (Figure S2c from Supplementary).

We further examined whether the phentolamine enhancement of macrolide activity could be extended to other Gram-negative bacteria (see Table 1). We, therefore, tested the susceptibilities of type strains of *S. typhimurium*, *K. pneumoniae*, *A. baumannii*, and *P. aeruginosa* (see Table 1). With the exception of *P. aeruginosa,* we observed a concentration-dependent reduction in macrolide MICs at phentolamine levels from 0 to 1/2 MIC. Additionally, for *S. typhimurium*, *K. pneumoniae,* and *A. baumannii,* 1/4 MIC phentolamine allowed 4–8 fold reductions in antibiotic use versus that that the macrolides used singly. At ½ MIC phentolamine, the macrolide MICs were further reduced 8-64 fold. (Figure 1c) These results demonstrated that phentolamine significantly enhanced the antibacterial activity of macrolide antibiotics against Gram-negative bacteria.

### 3.2. Efficacy of Phentolamine/Erythromycin Combinations for G. mellonella

These in vitro tests using phentolamine indicated robust synergy with the macrolides ERY, CLA, and AZM. We extended these observations and examined in vivo effects of phentolamine using the *G. mellonella* infection model. We utilized the most common clinical dosage for ERY at 30 mg/kg in the presence and absence of phentolamine at 0.02 mM/larvae using *E. coli* strain BW25113 for the infection challenge. Phentolamine and ERY monotherapies were ineffective in clearing the larval infections in 72 h. Survival in the phentolamine group was similar to the controls, while ERY monotherapy increased survival from 20 to 30% compared with controls. Challenges with *E. coli* 18FS resulted in survival for both phentolamine and ERY monotherapy groups that were the same as that of the controls. Interestingly, phentolamine/ERY combinations significantly increased *G. mellonella* survival from infections caused by both *E. coli* strains to 70% after 72 h (Figure 1d; Figure S1d from Supplementary).

### 3.3. Mechanism of Synergistic Activity of Phentolamine Combination with Macrolide Antibiotics

The success of the combination therapies using the in vivo model of infection motivated us to explore the mechanism of phentolamine synergism using *E. coli* strain BW25113 and ERY. The most probable modes of action for this synergism were reducing efflux pump activities and membrane damage.

**Figure 2:**
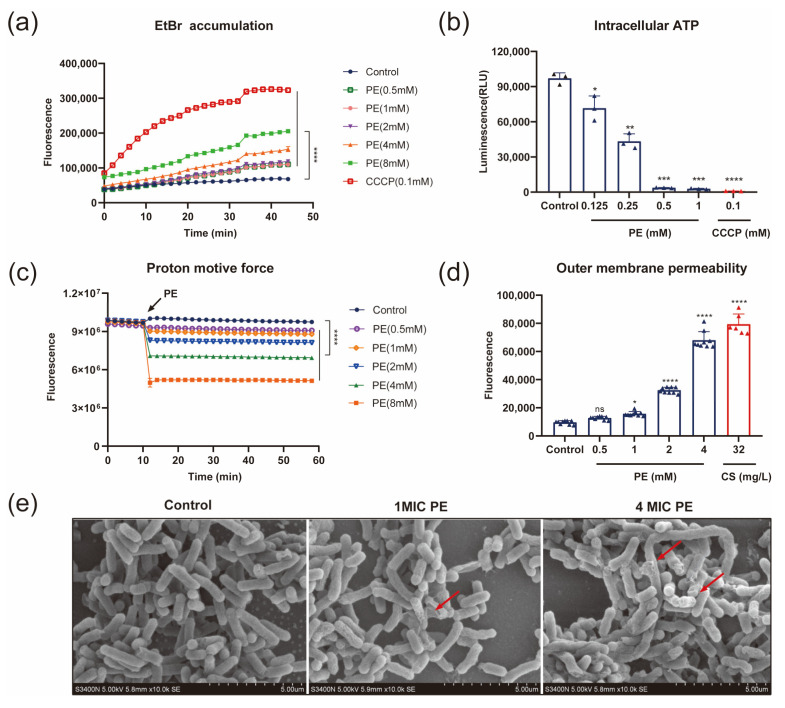
Phentolamine alters membrane permeability and efflux pump action. (**a**) EtBr accumulation in *E. coli* BW25113 treated with phentolamine. (**b**) Intracellular ATP levels of *E. coli* BW25113 were measured using a firefly luciferase-based ATP assay. (**c**) Measurements of ΔpH using the fluorescent dye BCECF-AM. (**d**) Phentolamine permeabilizes the outer membrane. Permeability was evaluated by measuring the fluorescence intensity of 1-N-phenylnaphthylamine (NPN). * *p* < 0.05, ** *p* < 0.01, *** *p* < 0.001, **** *p* < 0.0001). (**e**) Scanning electron micrographs of *E. coli* BW25113 cells treated with phentolamine. Morphological alterations (cell fragments and wrinkled surfaces) are indicated by the arrows. Scar bar, 5 µm.

Initially, we used the EtBr accumulation assay to examine efflux pump inhibition. We found that EtBr accumulation in the *E. coli* cells increased significantly after the addition of phentolamine, and this occurred in a concentration-dependent manner indicating that phentolamine inhibited efflux pump activity (Figure 2a). The function of the efflux pump requires ATP hydrolysis to provide energy, so we, therefore, measured intracellular ATP and ΔpH for in vitro cultures. We found that intracellular ATP levels decreased significantly after the addition of phentolamine in a concentration-dependent manner (Figure 2b). Moreover, we also evaluated the influence of phentolamine on the ΔpH using the fluorescence probe BCECF-AM. Phentolamine addition resulted in a distinct reduction in fluorescence intensity, indicating phentolamine had decreased or dissipated the pH gradient. We suspected that phentolamine reduced efflux pump activity via intracellular ATP and ΔpH reductions that would ultimately result in increased intracellular ERY accumulation. We further examined this by constructing strain BW25113Δ*tolc* that possessed an overall efflux pump defect due to the elimination of the TolC channel protein. The addition of 1/4 and 1/2 × MIC phentolamine to this strain resulted in MIC of 2- to 16-fold for ERY, CLA, and AZM. This indicated that phentolamine reduced efflux pump activity and increased the antibacterial activity of these macrolides by other mechanisms (Table 1). Additionally, we also found that the membrane permeability of the *E. coli* test strain was significantly increased in the presence of phentolamine (Figure 2d). The addition of phentolamine also directly damaged the outer membrane and resulted in cell fragmentation and wrinkling (Figure 2e). Taken together, these results demonstrated that phentolamine potentiates ERY activity by also damaging the outer membrane and, thus, would facilitate ERY cell entry.

**Figure 3:**
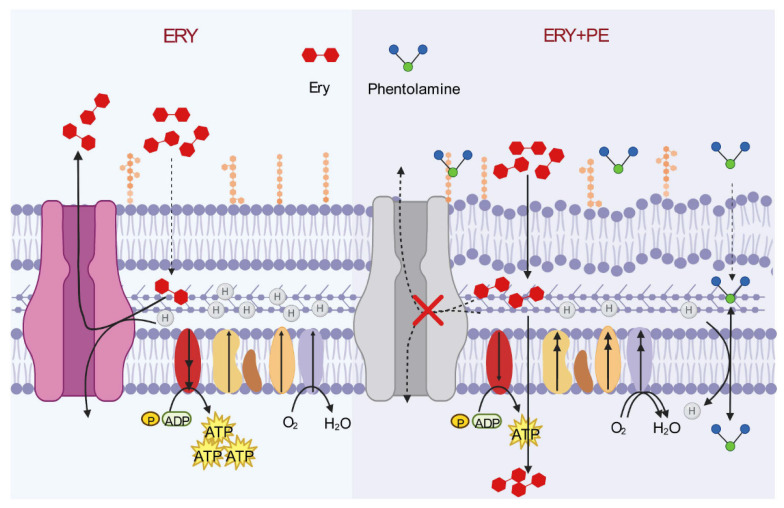
Proposed phentolamine mechanism of action. Phentolamine uncouples the electron transport chain, dissipates the PMF, and decreases ATP production to inhibit TolC-mediated efflux activity. Phentolamine also damages the outer membrane, and these activities enhanced ERY entry that could not be reversed by efflux pump activation. The sum of these effects are increased levels of macrolide potency against Gram-negative bacteria.

Combined with the above experimental results, we verified the previous hypothesis and clarified the mechanism of phentolamine enhancement of the antibacterial activity of macrolide antibiotics against *E. coli*. Erythromycin crosses the outer membrane but is expelled from the cell via PMF-dependent TolC-mediated efflux and, thus, cannot perform its role of inhibiting bacterial growth. Phentolamine uncouples the electron transport chain that dissipates the PMF and lowers ATP production and, thus, inhibiting TolC-mediated efflux activity. This is in addition to outer membrane damage caused by the compound. (Figure 3). Together our results indicated that phentolamine assists erythromycin entry into the *E. coli* cell and inhibits TolC-mediated efflux to enhance the antibacterial activity of erythromycin.

## 4. Discussion

Bacterial antibiotic resistance is one of the most serious modern threats to human and animal health [18], and MDR infections have severely limited treatment options [19]. In particular, Gram-negative pathogens are more difficult to treat due to their highly impermeable outer membranes that limit antibiotic accessibility [20,21]. Treatment of MDR infections often entails antibiotic combinations that expand the working antibacterial spectra, and this includes synergistic action [16,22]. Combination therapies rely on the inhibition of targets in the same and different pathways and the inhibition of the same target via differing mechanisms [23]. Macrolides are translation inhibitors that block nascent peptide exit from the large ribosomal subunit. These are among the most widely prescribed antibiotics, particularly for lung, skin, and soft tissue infections, due to their favorable safety and oral bioavailability profiles. However, their utility against Gram-negative bacteria is limited [24]. Therefore, the expansion of the antibacterial spectra of macrolides would greatly increase treatment options for MDR Gram-negative bacteria.

Phentolamine is used in the clinic by intravenous injection for the treatment of cardiovascular abnormalities, vasoconstriction, and erectile dysfunction by oral administration [25,26]. The strong vasodilation effect of phentolamine contraindicates its use for treatments other than topical applications. The results of the current study indicate that phentolamine and ERY can be used to topically treat skin or soft tissue infections, although treatment of more invasive infections is currently limited due to the decreased safety profile. Phentolamine has a dual mechanism of action against *E. coli;* as a membrane-penetrating agent and an efflux pump inhibitor. These actions facilitate macrolide entry into the cell, and its inhibitory effect on drug efflux further enhances antibiotic potency [27,28]. Therefore, the development of efflux pump inhibitors is an effective means to combat MDR bacterial infections, and, in particular, they have the potential to increase antibiotic effectiveness against Gram-negative pathogens [29]. For instance, colistin is often used clinically as a membrane penetrator in drug combinations. Unfortunately, colistin has strong renal toxicity, and resistance is now plasmid-borne in bacterial populations. These factors have limited its widespread use [30,31]. Phentolamine has been widely used in clinical practice, and a large number of studies and clinical data have confirmed its safety [32]. The current study is the first to describe its use as an antibacterial agent. Phentolamine significantly improved the antibacterial effect of macrolide antibiotics and expanded their antibacterial spectrum against MDR Gram-negative bacteria, and achieved a new use for an old medicine. The limitations of this study include the lack of larger numbers of other clinical MDR strains and the lack of a murine skin or soft tissue infection model. Additional studies are required to confirm the reproducibility of our results.

This study confirmed that phentolamine and macrolide antibiotic (erythromycin, clarithromycin, and azithromycin) combinations have significant synergistic bactericidal effects on MDR Gram-negative bacteria in vitro. The use of phentolamine effectively increased the MIC values of the macrolides. Importantly, phentolamine has a dual antibacterial effect by disrupting membrane permeability and inhibiting efflux pump activity. This synergism was also apparent in the *G. mellonella E. coli* infection model. Our findings constitute a promising alternative to combating MDR Gram-negative bacterial infections.

## Figures and Tables

**Table 1:** MICs of Erythromycin, clarithromycin, azithromycin, and phentolamine alone, MICs of Erythromycin, clarithromycin, azithromycin in the presence and absence of 1/4-1/2 MIC phentolamine.

			ERY (mg/L)			CLA (mg/L)			AZM (mg/L)		
Test Strains	Species	PE (mM)	ERY alone	1/4MIC PE	1/2MIC PE	CLA alone	1/4MIC PE	1/2MIC PE	AZM alone	1/4MIC PE	1/2MIC PE
BW25113	*E. coli*	1	32	8	2`	32	8	2	1	0.25	0.0625
ATCC 14028	*S. typhimurium*	2	32	8	2`	32	8	2	1	0.25	0.0625
ATCC 19606	*A. baumannii*	2	8	1	0.25	8	1	0.125	8	1	0.25
ATCC 27853	*P. aeruginosa*	4	128	64	64	128	128	64	32	32	16
ATCC 700603	*K. Pneumoniae*	2	128	32	16	128	32	16	16	4	1
BW 25113 ΔTolC	*E. coli*	1	1	0.5	0.125	2	1	0.0625	0.5	0.125	0.03125

## Data Availability

The authors confirm that the data supporting the findings of this study are available within the article and its Supplementary Materials.

## Supplementary Materials

The following supporting information can be downloaded at: https://www.mdpi.com/article/10.3390/antibiotics12040760/s1, Figure S1: (a) In vitro testing of phentolamine combined with 3 macrolide antibiotics (erythromycin, clarithromycin and azithromycin) indicated a synergistic action against *E. coli* strains with FICI values 0.5, 0.5 and 0.375. (b) The time–kill curves representing log10 changes in bacterial burden of *E. coli* BW25113 over 24 h, following exposure to phentolamine (1/2×MIC) in the presence of increasing clarithromycin and azithromycin concentrations (1/4 -1/2×MIC) are shown. (c) The time–kill curves representing log10 changes in bacterial burden of *E. coli* 18FS over 24 h, following exposure to phentolamine (1/2×MIC) in the presence of increasing erythromycin, clarithromycin and azithromycin concentrations (1/4 -1/2×MIC). (d) Phentolamine can improve the antibacterial activity of erythromycin against *E. coli* 18FS in vitro; Table S1: Bacterial strains, plasmids, and primers used in this study; Table S2: In vitro antimicrobial susceptibility profiles for test strain. MIC values of 11 antibiotics against *E. coli* 18FS. Gentamicin (GEN), Amikacin (AMK), Meropenem (MEM), Ceftaxime (CTX), Ceftazidime (CAZ), Cefoxitin (FOX), Ciprofloxacin (CIP), Florfenicol (FFC), Tetracycline (TET), Sulfamethoxazole/Trimethoprim (SMZ/TMP), Colistin (CS).
