# Supplementary material for: Phentolamine Significantly Enhances Macrolide Antibiotic Antibacterial Activity against MDR Gram-Negative Bacteria
Source: Antibiotics (Basel). 2023 Apr 14;12(4):760. doi: 10.3390/antibiotics12040760 (PMC10135019; doi:10.3390/antibiotics12040760)
Supplement: Supplementary file 1 [file antibiotics-12-00760-s001.zip › antibiotics-2304842-supplementary.pdf]

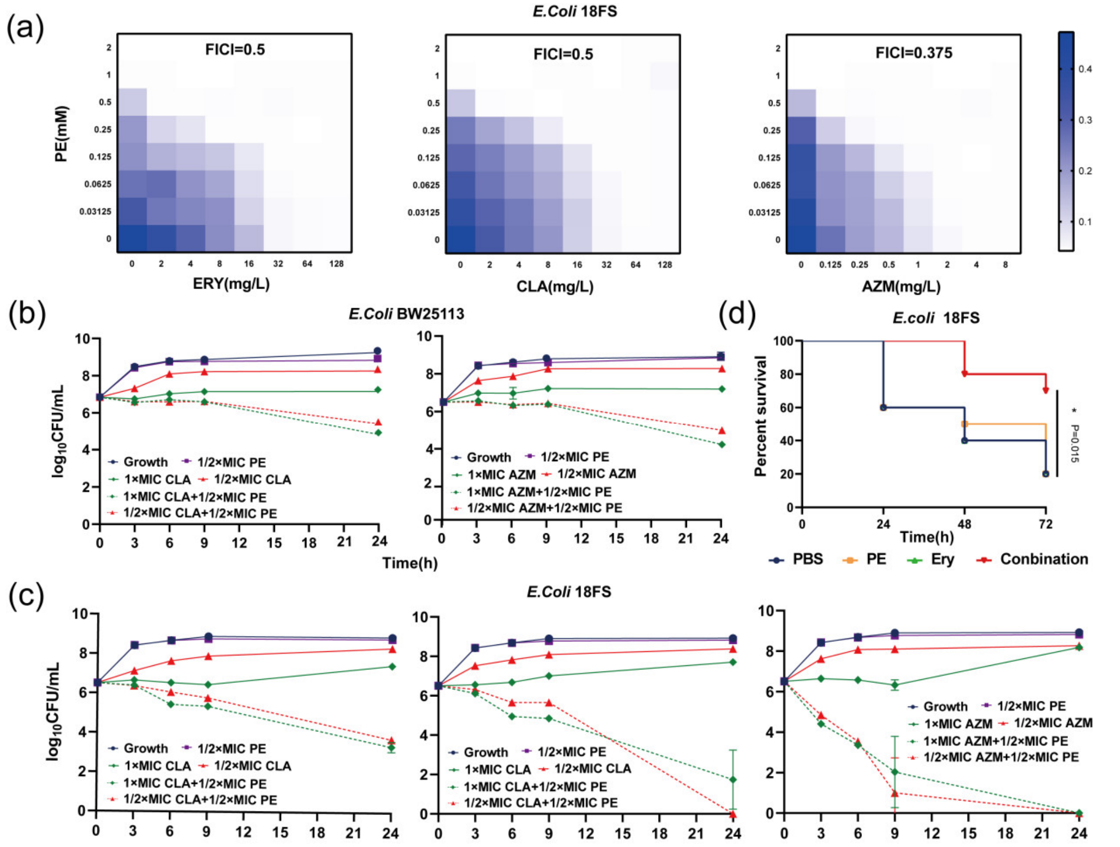

Figure S1 (**a**) In vitro testing of phentolamine combined with 3 macrolide antibiotics (erythromycin, clarithromycin and azithromycin) indicated a synergistic action against *E. coli* strains with FICI values 0.5, 0.5 and 0.375. (**b**) The time–kill curves representing log10 changes in bacterial burden of *E. coli* BW25113 over 24 h, following exposure to phentolamine (1/2×MIC) in the presence of increasing clarithromycin and azithromycin concentrations (1/4 –1/2×MIC) are shown. (**c**) The time–kill curves representing log10 changes in bacterial burden of *E. coli* 18FS over 24 h, following exposure to phentolamine (1/2×MIC) in the presence of increasing erythromycin, clarithromycin and azithromycin concentrations (1/4 -1/2×MIC). (**d**) Phentolamine can improve the antibacterial activity of erythromycin against *E. coli* 18FS in vitro.

Table S1. Bacterial strains, plasmids, and primers used in this study.

Strains, Plamids and Primers	Relevant Features and Use
pCasKP-apr	Bacterial expression of Cas9 nuclease and λ-Red system
PKD4	Bacterial expression; Gene knockout system
tolCF	5-TACAGTTTGATCGCGCTAAATACTGCTTCACCACAAGGAATGCAAGCAGCATTACACGTCTTGAG-3
tolCR	5-TACGTTGCCTTACGTTCAGACGGGGCCGAAGCCCCGTCGTCGTCAGCTGACATGGGAATTAGCCA-3

Table S2. In vitro antimicrobial susceptibility profiles for test strain. MIC values of 11 antibiotics against *E.coli* 18FS. Gentamicin (GEN), Amikacin (AMK), Meropenem (MEM), Ceftaxime (CTX), Ceftazidime (CAZ), Cefoxitin (FOX), Ciprofloxacin (CIP), Florfenicol (FFC), Tetracycline (TET), Sulfamethoxazole/Trimethoprim (SMZ/TMP), Colistin (CS).

		**MIC (mg/L)**										
**Test Strain**	**Species**	**GEN**	**AMK**	**MEM**	**CTX**	**CAZ**	**FOX**	**CIP**	**FFC**	**TET**	**SMZ/ TMP**	**CS**
18FS	*E. coli*	128	8	256	>256	>256	>256	256	256	256	>304/16	4
